# Developmental bias predicts 60 million years of wing shape evolution

**DOI:** 10.1073/pnas.2211210120

**Published:** 2023-05-01

**Authors:** Patrick T. Rohner, David Berger

**Affiliations:** ^a^Department of Biology, Indiana University, Bloomington, IN 47405-7107; ^b^Department of Ecology and Genetics, Uppsala University, Uppsala 752 36, Sweden

**Keywords:** developmental integration, developmental plasticity, fluctuating asymmetry, evolvability, macroevolution

## Abstract

The degree to which developmental biases affect trait evolution is subject to much debate. Here, we first quantify fluctuating asymmetry as a measure of developmental variability, i.e., the propensity of developmental systems to create some phenotypic variants more often than others, and show that it predicts phenotypic and standing genetic variation as well as deep macroevolutionary divergence in wing shape in sepsid flies. Comparing our data to the findings of a previous study demonstrates that developmental variability in the sepsid fly *Sepsis punctum* strongly aligns with mutational, standing genetic, and macroevolutionary variation in the Drosophilidae––a group that diverged from the sepsid lineage ca. 64 My ago. We also find that developmental bias in *S. punctum* wing shape aligns with the effects of allometry, but less so with putatively adaptive thermal plasticity and population differentiation along latitude. Our findings demonstrate that developmental bias in fly wings predicts evolvability and macroevolutionary trajectories on a much greater scale than previously appreciated but also suggest that causal explanations for such alignments may go beyond simple constraint hypotheses.

## Significance

Perturbation of development often has nonrandom effects on trait expression. Such developmental bias is thought to constrain adaptation by making evolution more likely to proceed in certain directions. Developmental biases can evolve, but what role natural selection plays in this process remains highly controversial. Here, we estimate developmental bias in insect wings and show that it predicts variation among individuals, the effects of mutation, as well as deep macroevolutionary divergence that unfolded over ~60 My. This suggests that developmental biases can be strong predictors of diversification on both short and long evolutionary timescales but also challenges the classic view of strong relationships between genetic variation and divergence as solely reflecting constraints on adaptation.

Development integrates environmental and genetic inputs to shape phenotypic outcomes. This integration is prone to produce some phenotypic variants more often than others. While such developmental variability, or bias, is a fundamental property of development (1–5), its role in evolution remains controversial. Because development can restrict the type of phenotypic variation that is generated via the mutational process, influencing the genetic and phenotypic variation available for selection to act upon, developmental bias has traditionally been viewed mainly as a constraining force in evolution (2, 6). At the same time, however, developmental variability is itself an evolved property and has, in principle, the capacity to respond to selection (7–9). Indeed, theory suggests that correlational selection on trait combinations can lead to the evolution of genetic architecture and to the buildup of genetic and developmental covariation (10–14). This in turn can bias the effects of future genetic or environmental perturbations in the direction favored by past selection (9, 13, 15, 16). Whether developmental bias facilitates or constrains adaptation might thus depend on whether past forces of selection reflect those of the present (9, 15, 17).

Developmental bias cannot be inferred from observed standing genetic or phenotypic variation, as such variation will have been directly influenced by selective removal of individuals expressing suboptimal trait combinations. Instead, it needs to be assessed by studying how genetic or environmental perturbations affect developmental outputs. One way of doing so is to study variation generated by de novo mutation, captured by the mutational variance–covariance matrix, **M**. Using this approach, Houle et al. (18) demonstrated that mutations cause nonrandom phenotypic variation, and that **M** in *Drosophila melanogaster* predicts macroevolutionary divergence across 40 My in Drosophilidae. This astonishing finding suggests strong developmental bias in the evolution of *Drosophila* wings but also poses fundamental questions with regard to the timescale on which developmental biases may shape evolutionary trajectories, and to what extent such patterns really reflect genetic constraints on adaptation (18, 19).

Although **M** provides information about how developmental biases structure mutational effects, **M** also captures variation caused by heterogeneous mutation rates across the genome. An alternative way of quantifying developmental variability in morphological traits is to measure fluctuating asymmetry between left and right homologs of paired bilateral structures. Because the left and right sides of the same organism share the same genome and environment, any difference between bilateral homologs are caused by developmental perturbations. The study of such fluctuating asymmetry therefore allows disentangling genetic and extrinsic environmental covariation from intrinsic *developmental* covariation (20–24). Here, we show that developmental bias (quantified by multivariate fluctuating asymmetry) in the dipteran wing predicts its evolution over the last 64 My. First, we demonstrate that the wing dimensions that show greatest developmental variability in the black scavenger fly *Sepsis punctum* are also those dimensions that possess most phenotypic and genetic variation and that show greatest macroevolutionary species divergence across Sepsidae. Second, we show that developmental variability in sepsids aligns with mutational, standing genetic, and macroevolutionary covariation in the distantly related Drosophilidae (18). Third, we demonstrate that the wing dimensions that show more developmental variability in *S. punctum* also show greater levels of sexual dimorphism and are strongly related to allometric scaling patterns. Finally, we show that, despite the strong alignment between developmental variability and macroevolutionary patterns, we find mixed support for an alignment between developmental bias and local adaptation to climate in *S. punctum*, a pattern that must have formed on a much shorter timescale. Taken together, our findings suggest that the phenotypic outcomes generated by developmental bias in dipteran fly wings may be nonrandom with respect to their fitness consequences and that simple constraint hypotheses may not be sufficient to fully explain alignments between measures of evolvability and deep macroevolutionary divergence.

## Results & Discussion

### Developmental Bias in Wing Shape.

To investigate the nature and degree of developmental variability, we first quantified fluctuating asymmetry in wing shape, captured by 11 two-dimensional landmarks placed at wing nodes (*SI Appendix*, Fig. S1). Analyzing all Procrustes shape variables simultaneously in a multivariate model, we found evidence for fluctuating asymmetry in the black scavenger fly *S. punctum* (Procrustes ANOVA, individual × side interaction: F_86,174_ = 27.15, *P* < 0.001, *SI Appendix*, Table S1) as well as its close relative *Sepsis fulgens* (individual × side interaction: F_95,192_ = 22.52, *P* < 0.001, *SI Appendix*, Table S1). Fluctuating asymmetry accounted for 8% and 9% of the total variation in wing shape (eta-squared, *η*^2^), respectively, after controlling for the effect of allometry (logarithmized centroid size). To test how similar the patterns of developmental variability are in the two species, we computed the developmental variance–covariance matrix, **D**, based on the shape component capturing fluctuating asymmetry. This matrix thus describes phenotypic changes induced by random developmental noise within individuals and represents our measure of developmental variability, or bias. We then compared **D** across the two species using a modified version of Krzanowski’s common subspace analysis following the method described in (25) (also see refs. 18 and 26). In brief, we compared the logarithmized variances of both matrices along the same set of orthogonal phenotypic dimensions of the wing. To limit bias in our estimates of effect sizes (25, 26), we chose to represent these phenotypic dimensions by the eigenvectors of an independently estimated third matrix––the phenotypic variance–covariance matrix, **P**––measured in *S. fulgens*. For consistency, we use this matrix as the reference to generate unbiased comparisons of different variance–covariance matrices throughout this study. However, we also repeated all comparisons by using other matrices as reference (see *SI Appendix*, Table S2) which showed that our conclusions do not depend on the matrix chosen as reference.

To make sure that **D** in *S. punctum* and *S. fulgens* was compared along subspaces in which there was statistically verified variation, we estimated the rank of the matrices by employing factor analytical modeling using ASReml-R (27). **D** in the two species were then compared along the first *k* dimensions of **P**, with *k* equal to the rank of the matrix of lowest rank. We applied this approach for any pair of variance–covariance matrices compared in this study (the number of dimensions used in each comparison varied between 9 and 12 and is reported in *SI Appendix*, Table S2). If the pattern of developmental variability is similar in the two species, we expect **D** in *S. punctum* and in *S. fulgens* to show similar amounts of variation along the eigenvectors of **P** estimated in *S. fulgens*. If so, we expect the variation captured by a given set of eigenvectors of **P** in one species to predict the variation along the same vectors in the other species. Using linear regressions of logarithmized variances along these vectors, we indeed find that the morphological variation generated by developmental variability is very similar in the two species (mean estimate and 95% confidence limits: coefficient of determination (r^2^) = 0.90 [0.81, 0.94], slope (b) = 0.65 [0.49, 0.78], number of dimensions used for comparison (n_Dim_) = 9; *SI Appendix*, Fig. S2). The total amount of variation captured by **D** (i.e., the trace of the variance–covariance matrix) was also similar (11.5 × 10^−5^ [9.9×10^−5^, 13.1 × 10^−5^] and 9.3 × 10^−5^ [8.3 × 10^−5^, 10.3 × 10^−5^] for *S. punctum* and *S. fulgens*, respectively, see *SI Appendix*, Fig. S2).

### Developmental Bias Predicts Phenotypic Variation, Evolvability, and Species Divergence across Sepsids.

**Fig. 1.**
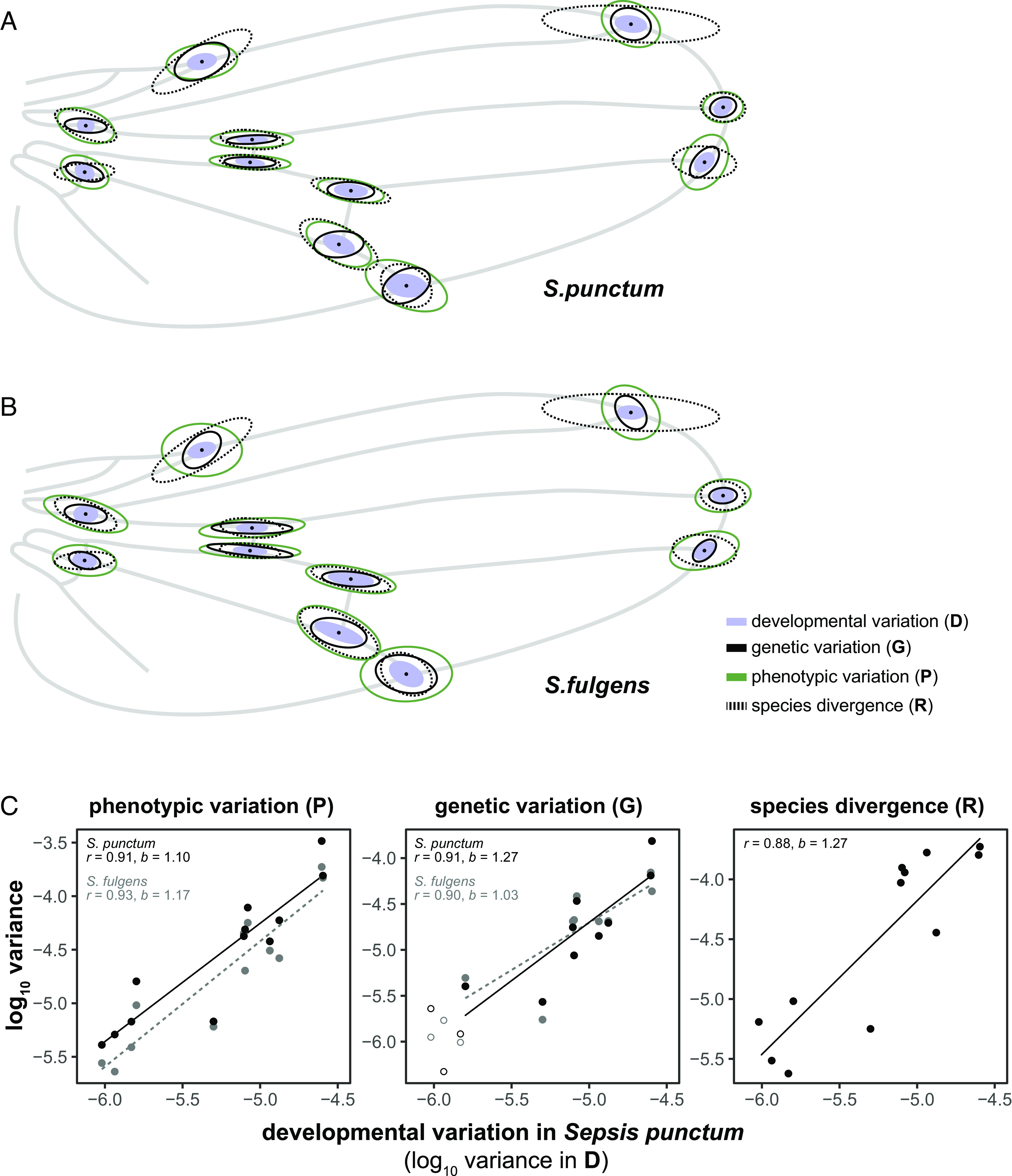
Ellipses representing variation in landmark positioning due to developmental variation within individuals (**D**, blue ellipses), phenotypic variation among individuals (**P**), broad-sense genetic variation (**G**), and evolutionary divergence (**R**) in *S. punctum* (*A*) and *S. fulgens* (*B*). Ellipses represent 7 SDs for better visibility of their orientation. (*C*) Variances of different covariance matrices are compared along the eigenvectors of **P** in *S. fulgens.* Because REML-estimated covariance matrices differed in the number of ranks, slopes and correlations were calculated based on the first 9 (**G**) or 12 (**P, R**) dimensions of the **P** matrix in *S. fulgens*.

Next, we investigated whether developmental variability predicts phenotypic variation observed among individuals (i.e., the **P** matrix). Using the common subspace analysis described above, we found that **D** is closely related to **P** in *S. punctum* (r^2^ = 0.83 [0.74, 0.86], b = 1.10 [0.98, 1.21], *P* < 0.001, n_Dim_ = 12, Fig. 1). This result shows that phenotypic variation among individuals reflects the same morphological changes that are produced by developmental variability within individuals, indicating that your multivariate measure of fluctuating asymmetry indeed captures developmental bias. Next, we asked whether **D** is also aligned with standing genetic covariation, captured in the broad-sense genetic variance–covariance matrix **G**, estimated from the variability among isofemale lines reared in a common garden experiment (28, 29). Although we restricted our analysis to only the first nine dimensions (the rank of **G**), we found a strong alignment between **D** and **G** in *S. punctum* (r^2^ = 0.83 [0.74, 0.86], b = 1.27 [1.02, 1.47], *P* < 0.001, n_Dim_ = 9, Fig. 1). Strikingly, we found a similar relationship comparing **D** in *S. punctum* to **G** estimated from isofemale lines of *S. fulgens* (30) (r^2^ = 0.81 [0.71, 0.86], b = 1.03 [0.77, 1.25], *P* < 0.001, n_Dim_ = 9, Fig. 1), showing that this relationship extends across species.

Next, we tested whether developmental variability relates to macroevolutionary divergence in Sepsidae. The evolutionary variance–covariance matrix (**R**) based on wing shape of 36 different taxa across 8 different sepsid genera, encompassing most major clades of Sepsidae, also aligned closely with **D** (r^2^ = 0.77 [0.66, 0.83], b = 1.27 [1.10, 1.41], *P* < 0.001, n_Dim_ = 12, Fig. 1 and *SI Appendix*, Fig. S3). Notably, the slopes of the logarithmized relationship between **D** and **G** or **R** are close to unity, suggesting that there is a strong proportional relationship between developmental variability, evolvability, and the rate of species divergence in wing shape, as would be expected under a scenario where developmental biases exert fundamental constraints on macroevolutionary trajectories.

### Developmental Bias in Sepsid Flies Predicts Mutational, Standing Genetic, and Macroevolutionary Variation in Drosophilids.

**Fig. 2.**
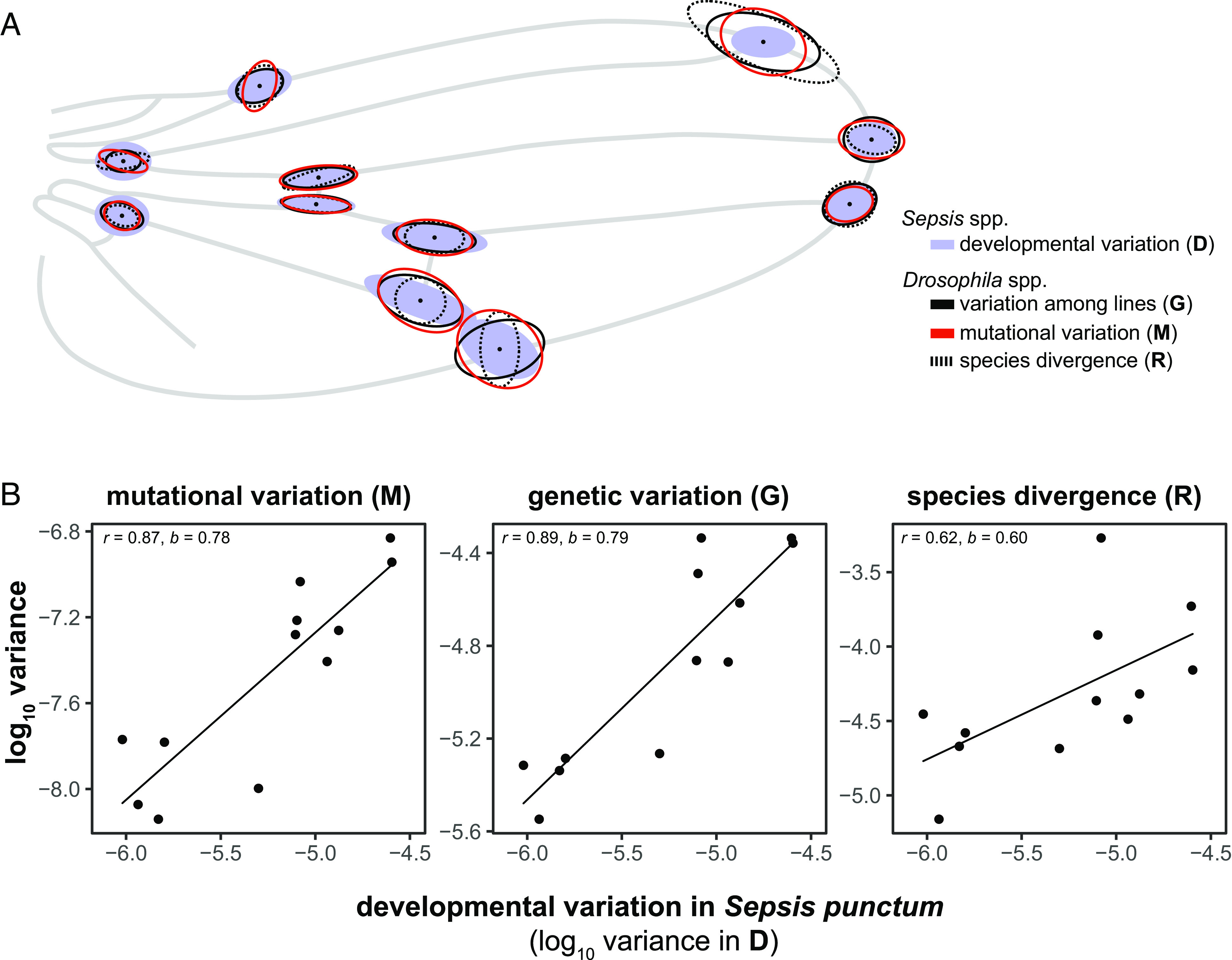
(*A*) Relationship between developmental bias (**D**) in *S. punctum* (blue ellipses) and variation due to mutational input (**M**), genetic variation (**G**), and macroevolutionary divergence (**R**) in drosophilids (data from ref. 18). To better illustrate the direction of shape variation, all variance–covariance matrices were scaled to the same (arbitrary) size. The areas of ellipses are relative to the total variance within each matrix. (*B*) The variance in **D** and the respective matrix of comparison along the first 12 eigenvectors of the **P** matrix in *S. fulgens*.

Our results recapitulate the striking findings in drosophilids where the mutational matrix, **M**, predicts divergence across 40 My (18). To test how deeply conserved the observed developmental bias is, we compared our estimates of **D** in sepsid flies to the covariation in fruit fly wings presented by Houle et al. (18). **D**, estimated in *S. punctum*, predicts **M** (r^2^ = 0.76 [0.61, 0.83], b = 0.78 [0.66, 0.88], *P* < 0.001, n_Dim_ = 12, Fig. 2) and **G** (r^2^ = 0.79 [0.69, 0.85], b = 0.79 [0.70, 0.86], *P* < 0.001, n_Dim_ = 12, Fig. 2) in *D. melanogaster*, as well as macroevolutionary wing shape divergence across the Drosophilidae (r^2^ = 0.38 [0.28, 0.48], b = 0.60 [0.50, 0.69], *P* < 0.001, n_Dim_ = 12, Fig. 2). These alignments are striking considering that the common ancestors of Sepsidae and Drosophilidae diverged around 64 Mya (31) [for comparison, the divergence time between sepsids and drosophilids is similar to that between elephants and hyraxes, estimated to be 66.7 My (32)].

### Development as a Biasing Factor across Levels of Biological Organization.

**Fig. 3.**
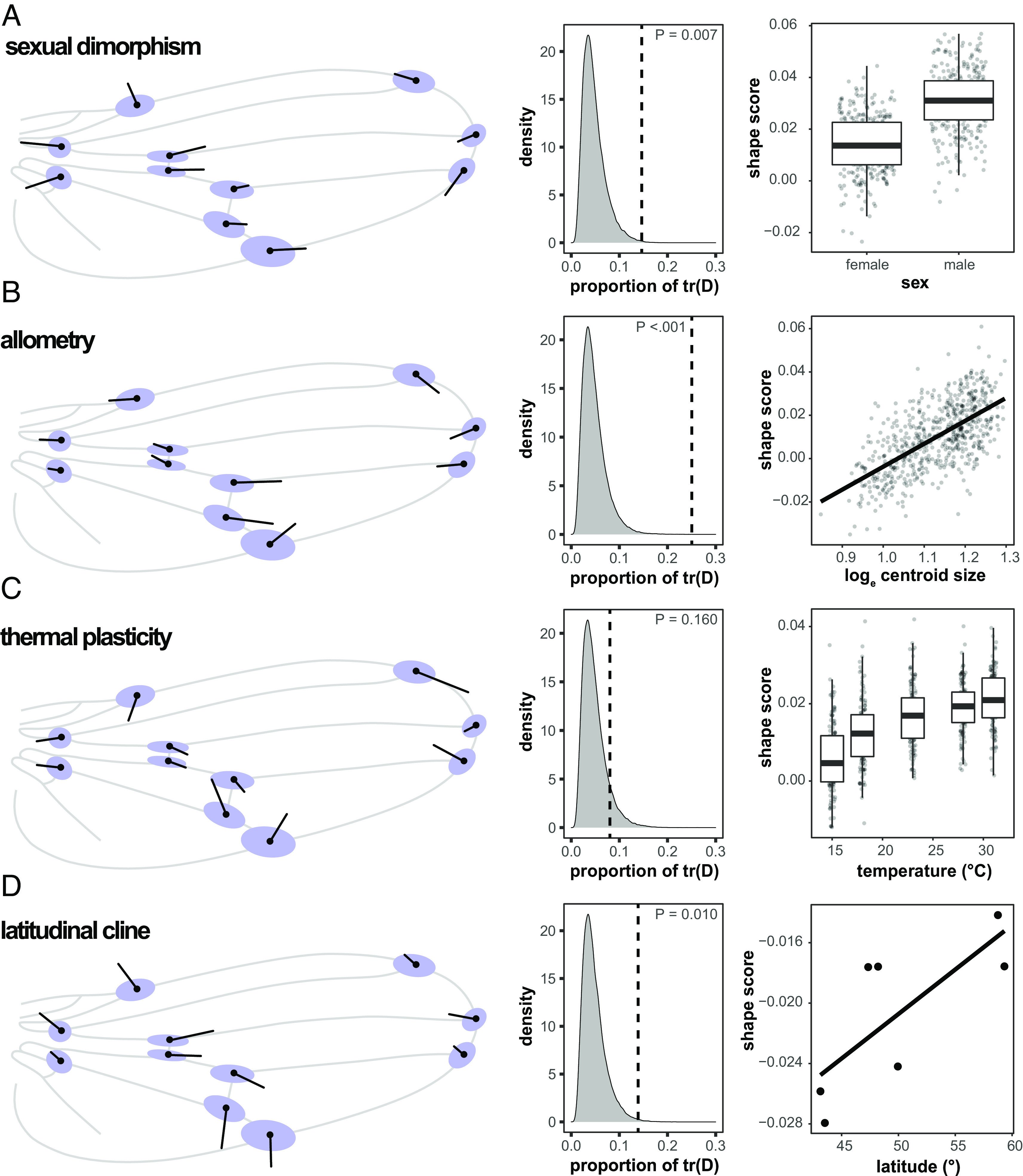
Alignment between developmental covariance (**D**, indicated in blue) and shape deformation vectors due to: (*A*) sexual dimorphism, (*B*) allometry, (*C*) thermal plasticity, and (*D*) latitudinal population differentiation. Density plots show the proportion of developmental variance [tr(D)] captured by the observed shape vector (indicated with a hatched line) relative to a randomized sample of the shape vector (n = 10,000 replicates). Plots on the right indicate how shape scores [i.e., projections of the shape data onto the corresponding shape deformation vector (42)] vary with the corresponding variable.

Developmental variation in wing shape is clearly anisotropic, conserved across two sepsid species, and correlated with both macroevolutionary species divergence and mutational effects in *Drosophila*. At face value, these strong alignments are consistent with the presence of fundamental constraints dictated by the developmental architecture governing fly wing evolution. Indeed, a large body of literature documents developmental coordination of *Drosophila* wings (e.g., refs. 33 and 34–35). One source of this coordination may be the way adult wings form from imaginal discs. The dorsal and ventral sides of the future wing blade, including the dorsal and ventral longitudinal proveins, form independently next to each other before they establish physical contact during folding and evagination. The precise positioning of the longitudinal wing veins therefore needs to be tightly controlled to align during evagination (35, 36). In contrast to longitudinal veins, the two crossveins form later during development once ventral and dorsal surfaces have been aligned (36). This difference in timing may explain why the positioning of landmarks relating to crossvein positioning is more developmentally variable relative to other landmarks in the wing and why this variation is focused along the proximo-distal axis (Fig. 1). Consistent with this hypothesis, recent evidence suggests that variation in wing morphology generated by genetic or environmental perturbations is constrained to fall along a restricted set of phenotypic dimensions (37–39). To test whether these biases also are reflected at other levels of biological organization in sepsid flies, we assessed whether our estimate of **D** aligns with sexual dimorphism and allometry in wing shape in *S. punctum*. There was more variation in **D** along both shape deformation vectors than expected by chance (sexual dimorphism: *P* = 0.007; allometry: *P* < 0.001). For sexual dimorphism, 15% of the total variation in **D** (given by the matrix trace) was found along the shape vector, compared to 5% (on average) along randomized vectors (Fig. 3*A*). For allometry, as much as 24% of the variation in **D** was captured by the shape vector (Fig. 3*B*), demonstrating that developmental variability recapitulates two major sources of phenotypic variation (size and, to lesser extent, sex) observed within natural populations of sepsids (40, 41).

### Can Developmental Constraints Explain the Alignments?

At first glance, the strong relationship found between **D**, **P**, **G**, and **R** could be interpreted as supporting constraint hypotheses to best explain sepsid wing shape evolution. A prediction under this hypothesis is that **D** also should be aligned with evolution on shorter time scales. To evaluate this prediction, we tested whether **D** aligns with population differentiation along latitude and thermal plasticity in *S. punctum* wing shape. These patterns have previously been argued to represent local adaptation to climate as the direct effect of rearing temperature on wing shape is recapitulated in the genetic differences found among high- and low-latitude populations (28) (Fig. 3 *C* and *D*). Interestingly, we found no significant alignment between thermal plasticity and **D** (9% of the total developmental variation along the plasticity vector, compared to 5% along a randomized vector, *P* = 0.160, Fig. 3*C*) and a moderate 14% of the total developmental variation along the shape vector for latitude (*P* = 0.010, Fig. 3*D*). Closer inspection of these relationships shows that there is comparatively little agreement between **D** and the effects of temperature and latitude as the latter mostly relate to the relative width of the wing (Fig. 3 *C* and *D*). Relative wing width (i.e., aspect ratio) has previously been linked to flight capacity (43) and climate adaptation (44) in *Drosophila*. Hence, these results suggest that at least some aspects of functional wing shape variation can evolve relatively free of developmental constraints in *S. punctum*.

**Fig. 4.**
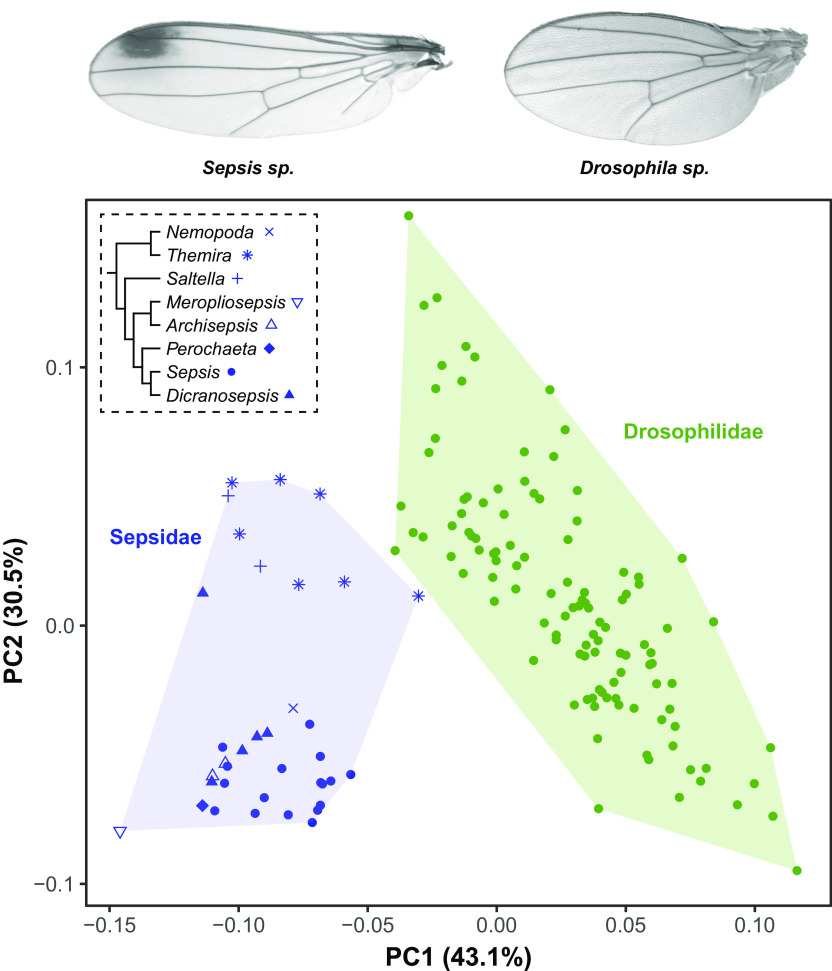
Macroevolutionary morphospace showing interspecific variation among sepsid (blue) and drosophilid (green) wing shapes. While sepsids and drosophilids differ in overall wing shape, the disparity in Sepsidae (Procrustes variance = 5.88 × 10^−3^) is not significantly different from that observed in drosophilids (Procrustes variance = 5.00 × 10^−3^). Within Sepsidae, different clades (indicated by symbols) occupy different areas in the morphospace, indicating a strong phylogenetic signal in wing shape. The phylogenetic relationship among genera is indicated in a cladogram following (45). Data for Drosophilidae are from Houle et al. (18).

Indeed, quantitative characters are typically not expected to be strongly influenced by genetic constraints over extended evolutionary time frames, as studied here, due to their polygenic basis and large mutational target sizes. In line with this expectation, the study on drosophilids by Houle et al. (18) found an almost perfect proportionality between **M** and **R** coupled with a high phylogenetic heritability, but a rate of divergence much lower than predicted under drift given the estimated mutational variance. Therefore, a lack of mutational input seems unlikely to explain the alignment between **M** and **R** in drosophilid wing evolution (18). Here, studying sepsid flies––a group of flies that diverged from drosophilids 64 Mya––we similarly report proportionality between **D**, **G**, and **R**. The degree of morphological disparity in sepsids (Procrustes variance = 5.88 × 10^−3^) and drosophilids (Procrustes variance = 5.00 × 10^−3^) does not differ significantly (Fig. 4; difference between Procrustes variances: 8.84 × 10^−4^, Z = 0.31, *P* = 0.403), suggesting that the rate of divergence is similar for the two clades. Thus, assuming that mutation rates are not fundamentally different in the two clades, and given that mutational input is unlikely to explain wing shape evolution in drosophilids, the developmental bias quantified here would seem unlikely to pose hard constraints on the evolution of dipteran wing shape.

### Correlational Selection Acting on Multiple Levels of Biological Organization.

How do we reconcile the alignment between developmental bias and macroevolutionary trajectories with seemingly abundant genetic variation available for evolution? An alternative explanation for the observed alignments emerges if we consider that different dimensions of the wing may experience different strengths of directional and stabilizing selection, such that developmental bias has itself evolved to align with the fitness surface (7, 9, 10, 46). Under this scenario, proportionality between **D**, **M**, and **R** is observed, not because development constrains macroevolutionary rates, but because pervasive correlational stabilizing selection has restricted developmental variability, mutational effects, and divergence to all occur along the same phenotypic dimensions. How common and on which evolutionary time frames such processes can occur is still debated. For example, some recent models highlight that correlational selection can reshape **M** on relatively short timescales in specific scenarios [e.g., high rates of mutational input (13), large drift load (47, 48), or high levels of maladaptive gene flow (49)], while other models suggest that mutational and developmental biases evolve to align with the forces of correlational selection under fairly restrictive conditions (50–53). Another route by which correlational selection could indirectly shape **M** and cause it to align with **R** is via selection for robustness to stress imposed by the environment. The conditions under which selection can lead to the evolution of such environmental canalization are more permissive because environmental variation is typically frequent and persistent (53). Similarly, selection for adaptive developmental plasticity may facilitate more mutational variation along the phenotypic dimensions that are responsive to the environmental variation (54, 55). This hypothesis is in line with the strong alignment found between developmental bias and allometry in *S. punctum* (Fig. 3*B*), which shows pronounced, adaptive developmental plasticity in adult body size in response to highly variable food supply during larval development (56). It is thus possible that evolution of **D** and **M** results as a by-product of selection for adaptive responses to environmental variation (55, 57, 58). An expectation under this scenario is that environmental and mutational variation tend to have similar effects on phenotypic expression. However, even though the effects of environmental perturbations and mutation have been found to sometimes align, this may not to be the case more generally (59–61). Future work will be necessary to explore to what degree developmental biases are shaped by past and present forces of selection.

## Conclusions

Mutations are recognized as the ultimate source of genetic variation and evolvability, but it is via developmental processes that nucleotide changes cause heritable variation in phenotypes. Here, we measured fluctuating asymmetry to assess variability in developmental processes and show that this bias shapes **M** and **G** and strongly correlates with macroevolutionary diversification. Alignments between standing genetic variation within populations and macroevolutionary rates have previously been observed for other morphological features in plants, insects, lizards, fish, and other vertebrates (62–65), suggesting that the patterns reported here for dipteran wing shape may highlight a more general phenomenon. Our results are consistent with the notion that, while microevolutionary patterns may reflect the forces of natural selection, macroevolution may often unfold by drift along genetic lines of least resistance delineated by the architecture of the developmental system (66). However, our findings add to a growing body of literature suggesting that these macroevolutionary patterns may in fact reflect the forces of stabilizing correlational selection, exercising a similar influence on both developmental architectures and species divergence. To what extent genetic architecture and developmental systems can evolve by natural selection is still a controversial question in need of further theoretical and empirical attention. Indeed, the consequences for rates of adaptation over both short- and long-term evolutionary scales depend on whether past forces of natural selection reflect those of the present, and when not, whether contemporary selection may reshape developmental biases set by previous adaptations. Irrespective of whether our results are best explained by constraints or selective forces (or a combination thereof), the revealed alignments between developmental bias and deep divergence are striking, challenging the classic view of strong relationships between evolvability and divergence as solely reflective of constraints.

## Methods

We combined previously published data on intraspecific and interspecific variation in sepsid wing shape with the data we generated on fluctuating asymmetry in *S. punctum* and *S. fulgens* (see below). Wings were dissected from killed flies and embedded in a standardized amount of Euparal on glass slides. Slides were photographed using a LeicaDFC490 camera mounted on a Leica MZ12 microscope. To quantify wing shape, we digitized 11 homologous two-dimensional landmarks (*SI Appendix*, Fig. S1) using TpsDig2 (67). Landmarks were aligned to Houle et al.'s (18) consensus configuration using a Procrustes analysis in *MorphoJ* (68). Our final dataset contained measurements of 11 two-dimensional landmarks for a total of 3,204 sepsid wings.

### Fluctuating Asymmetry in Wing Shape.

To estimate **D**, fluctuating asymmetry (FA) in wing shape was assessed in an outbred laboratory population of *S. punctum* (originally collected in Zurich, Switzerland; n = 87 male individuals). We measured the wing shape of the left and right wings of each individual twice to account for measurement error (rendering a total number of 348 measurements for each of the 22 landmark coordinates). We only considered males to estimate asymmetry. Because FA in wing size (e.g., ref. 69) is likely to contribute to FA in shape, we first removed any allometric variation from our FA dataset by extracting the residual variation from a multivariate regression of Procrustes coordinates onto logarithmized centroid size. The statistical significance of FA in wing shape was assessed by estimating the effect of individual identity, side (left or right), and the individual-by-side interaction using a Procrustes ANOVA (*SI Appendix*, Table S1). The individual-by-side interaction (i.e., fluctuating asymmetry) was tested against the individual-by-side-by-measurement interaction as an error term. The FA component, i.e., the specimen-specific deviation from symmetry adjusted for directional asymmetry, was then quantified using the function *bilat.symmetry*() in the R-package *geomorph* (70). The variance–covariance matrix of this component then describes the correlated shape changes due to random developmental fluctuations in one side of the organism (i.e., **D**). The entries of **D** represent the contribution of one side to the total variance and covariance. We did not find any evidence for antisymmetry. In addition to the Procrustes ANOVA, we also employed factor analytical mixed models in ASReml-R (27) to generate restricted maximum likelihood estimates of the developmental variance–covariance matrix. Specifically, we fitted wing shape variables as a function of the fixed effects of individual and side and estimated the variance–covariance matrix for the individual-by-side interaction (random effect). All coordinates were multiplied by 10,000 before analysis to ease model convergence. The factor analytical mixed model and Procrustes ANOVA rendered similar estimates of **D** but the former allowed us to further assess the dimensionality of **D** (and all other estimated matrices that were later compared; see *Comparing Variance–Covariance Matrices* below).

We also used a factor analytical mixed model to estimate the phenotypic variance–covariance matrix (**P**) based on the same dataset by adding side as the only fixed effect (to account for directional asymmetry) and individual identity as random effect. We followed the same procedure to generate the corresponding **D** and **P** matrices for *S. fulgens* (also originally collected in Zurich, Switzerland; n = 96 male individuals).

### Standing Genetic Variation in Wing Shape.

To estimate **G**, we quantified variation among isofemale lines of several geographic populations of *S. punctum* (7 populations, 74 lines) and *S. fulgens* (9 populations, 42 lines) reared in two separate common garden experiments that manipulated rearing temperatures (*S. punctum*: 15, 18, 23, 28, 31 °C; *S. fulgens*: 12, 18, 24, 30 °C) in both sexes (28–30). Again, we employed factor analytical models to generate restricted maximum likelihood estimates of the genetic variance–covariance matrix **G** using ASReml-R. Sex and rearing temperature were added as fixed effects, while population and isofemale line were used as random effects. Because genetic variances are based on isofemale lines, the resulting **G** matrix represents broad-sense estimates of genetic variances and covariances.

### Species Divergence in Wing Shape.

To estimate **R**, the matrix of variances and covariances of trait change with divergence in a Brownian motion, we measured the wing shapes of wild-caught and laboratory-reared specimens of 36 different species (and subspecies) of sepsid flies (40, 71, 72). We computed the matrix **R** based on the inverse of the relationship matrix among taxa [S^−1^, (73)] using a mixed model in ASReml-R. Due to the lack of a published dated phylogeny that contains branch length information for all species with morphological data, we combined the topologies of two published phylogenies (45, 74) to generate a concatenated cladogram. We used Grafen’s (75) method (which scales node height relative to the number of descendant tips minus one) to compute branch lengths. Repeating the analysis with all branch lengths set to unity or without taking the phylogenetic relationship into account (i.e., adding species identity as random effect) produced qualitatively very similar estimates of **R**.

### Data from the Drosophilidae.

To compare **D** to covariation in drosophilids, we extracted the (homozygous) mutational (**M**), genetic (**G**), and macroevolutionary (**R**) covariance matrices for *D. melanogaster* from (18). Morphological disparity in wing shape (as measured by Procrustes variance) was compared between Sepsidae and Drosophilidae using the function *morphol.disparity()* as implemented in *geomorph*.

### Estimating the Dimensionality of Covariance Matrices.

Although all our analyzed matrices contain 18 dimensions (due to the loss of four dimensions for scaling, rotation, and positioning during Procrustes analysis), geometric morphometric covariance matrices are often rank deficient due to redundant covariance among landmark variables (76). To assess how many dimensions of **D**, **P**, **G**, and **R** have statistical support, we fitted reduced-rank factor analytic mixed models in ASReml-R, with the same structure for fixed and random effects as described above. For each matrix, we compared models of different ranks based on Akaike’s Information Criterion (AIC). Specifically, we started with a rank of one and continually increased the number of dimensions until model fit was not significantly increased (ΔAIC < 2) or models did not converge. We then extracted the reduced rank variance–covariance matrices (**D, P, G,** and **R**) from these best-fitting models for further analysis using the R package ASExtra4 (77, see *SI Appendix*, Table S3). Error variances were estimated separately for each shape variable in all models.

### Comparison of Variance–Covariance Matrices.

Even though we focus on comparisons between **D** in *S. punctum* and our other variance–covariance matrices of interest, we compare the variances of these matrices along the eigenvectors of **P** estimated in *S. fulgens* to minimize bias in the estimates of regression slopes (25). Following (25, 26), we decomposed the **P** matrix estimated in *S. fulgens* into its eigenvectors $K_{D}$ and calculated the variance along $K_{D}$ for each of the respective variance–covariance matrices of interest as the diagonal entries of the matrix $K_{D}^{T}XK_{D}$ , where T denotes transposition and **X** refers to the matrix being compared (**D** and **P** for *S. punctum and S. fulgens,* respectively; **G** for *S. punctum*, *S. fulgens*, and *D. melanogaster;* **R** for Sepsidae and Drosophilidae; **M** for *D. melanogaster*). We then calculated coefficients of determination (r^2^) and slopes (b) between these logarithmized variances for a given matrix (e.g., **R** in Drosophilidae) and the corresponding logarithmized variances of **D** for *S. punctum*. To avoid comparing matrices along null spaces with deficient variance, each matrix pair was compared along only the first *k* dimensions of **P,** with *k* equal to the rank of the matrix with lowest rank (9 for comparisons between **D** and **G** matrices, 12 for all other comparisons).

To provide approximate 95% confidence limits around correlations and slopes, we resampled the variance–covariance matrices from the factor analytical models with best support (based on AIC), using the REML-MVN approach (78). This approach uses asymptotic resampling of REML estimates, taking advantage of the fact that the sampling distribution of variance–covariance matrices is well approximated by a multivariate normal distribution at large sample size. We performed the MVN resampling on the “G-scale” using the *mvtnorm* package for R (also see: refs. 62 and 63). With this approach, we resampled 10,000 matrices of each kind and subjected them to the common subspace analysis.

### Comparing **D** to the Directions of Multivariate Phenotypic Plasticity and Genetic Differentiation.

To test whether **D** in *S. punctum* is aligned with latitudinal differentiation among *S. punctum* populations observed in ref. 28, we computed the average population mean shape across lines, sexes, and rearing temperatures and used a multivariate regression of population mean shape onto logarithmized centroid size and latitude of origin to extract the partial regression coefficients associated with the latitudinal shape change vector as:

$$Y=\beta_{0}+{CS*\beta }_{\mathrm{CS}}+{Lat*\beta }_{\mathrm{Lat}}+\varepsilon ,$$

where Y represents the matrix of Procrustes shape variables (averaged by population), $\beta_{0}$ is the vector of intercepts, $\beta_{\mathrm{CS}}$ is the partial regression coefficient of log centroid size, $\beta_{\mathrm{Lat}}$ refers to the effect of latitude, and ε is the residual error term. Similar coefficients for the effects of allometry, sexual dimorphism, and temperature were calculated by fitting wing shape averaged by isofemale line as a function of log centroid size, population, sex, and rearing temperature as:

$$Y=\beta_{0}+{CS*\beta }_{\mathrm{CS}}+Sex*\beta_{\mathrm{Sex}}+Pop*\beta_{\mathrm{Pop}}+Tem*\beta_{\mathrm{Tem}}+\varepsilon ,$$

We then quantified the amount of developmental variability in the direction of each shape vector as:

$$e_{\beta }=\;\frac{\beta^{T}D\beta }{{\left|\beta \right|}^{2}},$$

where β is the shape deformation vector of interest and *T* denotes transposition. This measure ( $e_{\beta }$ ) gives the amount of developmental variation in the direction of the shape vectors $\beta_{\mathrm{CS}}$ , $\beta_{\mathrm{Sex}}$ , $\beta_{\mathrm{Tem}}$ , or $\beta_{\mathrm{Lat}}$ [this is thus an allegorical approach to that used to estimate evolvability as the amount of genetic variation available along the direction of multivariate selection (79)]. If developmental variability occurs primarily along the axis of *β*, we expect $e_{\beta }$ to be large (i.e., close to the first eigenvalue of **D**). To compute a more intuitive effect size, we calculated the ratio of $e_{\beta }$ and the total developmental variance (i.e., the trace) of **D.** To test whether $e_{\beta }$ is larger than expected by chance, we conducted a randomization test by reshuffling the observed shape deformation vectors 10,000 times and recalculating e for this random set of *β’s*.

## Supplementary Material

Appendix 01 (PDF)

Dataset S01 (CSV)

Dataset S02 (CSV)

Dataset S03 (CSV)

Dataset S04 (CSV)

Dataset S05 (CSV)

Dataset S06 (CSV)

Dataset S07 (CSV)

Dataset S08 (CSV)

Dataset S09 (CSV)

Dataset S10 (CSV)

Dataset S11 (CSV)

## Data Availability

Morphological data underlying the analysis of fluctuating asymmetry and species divergence in sepsid flies, as well as the computed reduced-rank REML variance-covariance matrices are attached as supplementary datasets.).
