# Supplementary material for: Developmental bias predicts 60 million years of wing shape evolution
Source: Proc Natl Acad Sci U S A. 2023 May 1;120(19):e2211210120. doi: 10.1073/pnas.2211210120 (PMC10175750; doi:10.1073/pnas.2211210120)
Supplement: Supplementary file 1 — Appendix 01 (PDF) [file pnas.2211210120.sapp.pdf]

PNAS

Supporting Information for
Developmental bias predicts 60 million years of wing shape evolution

Patrick T. Rohner & David Berger

Patrick T. Rohner
Email: prohner@iu.edu

This PDF file includes:

Figures S1 to S3
Tables S1 to S3

Other supporting materials for this manuscript include the following:

Datasets S1 to S10

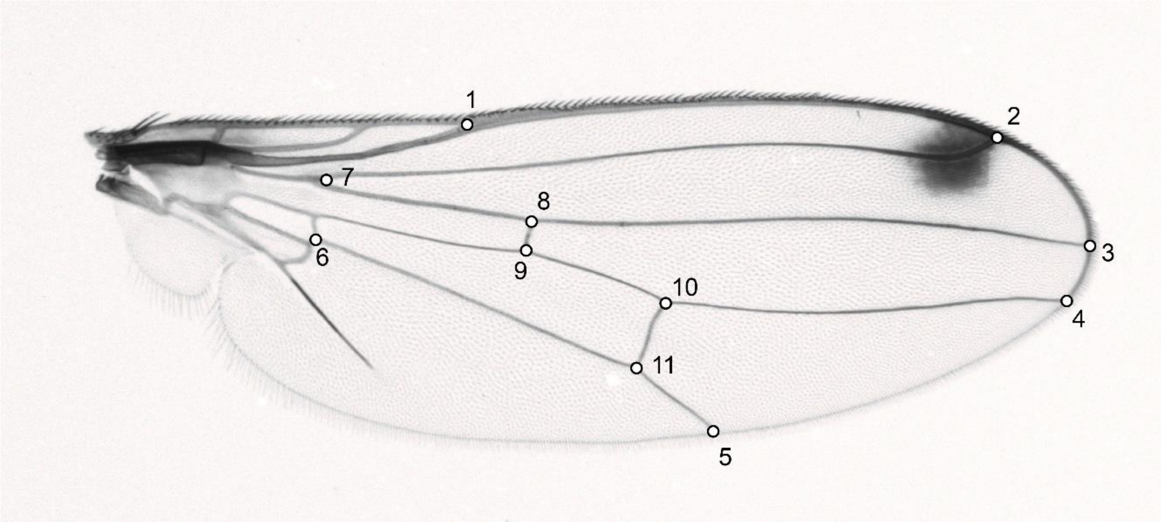

Fig. S1. Position of the 11 two-dimensional landmarks used in this study.

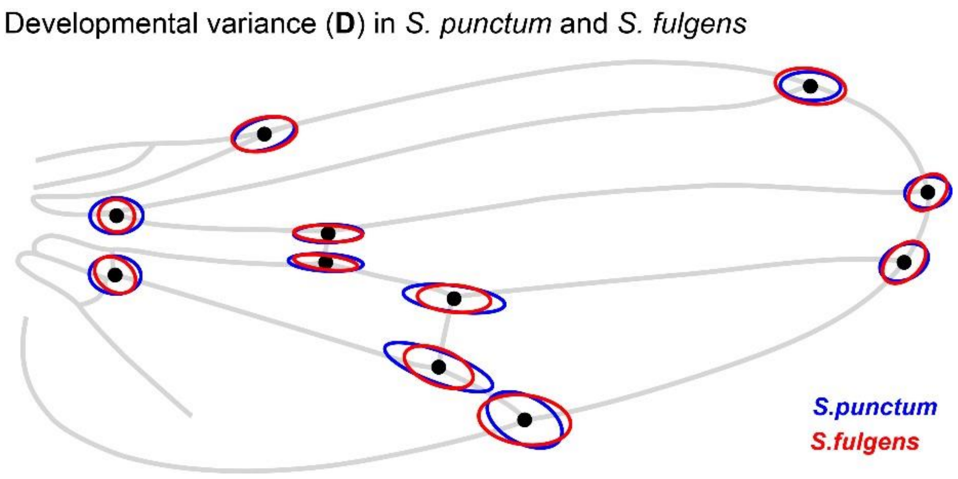

Fig. S2. The developmental variation (**D**) is very similar in direction and comparable in size in *S. punctum* and *S. fulgens*. **D** for both species has been multiplied by for illustration.

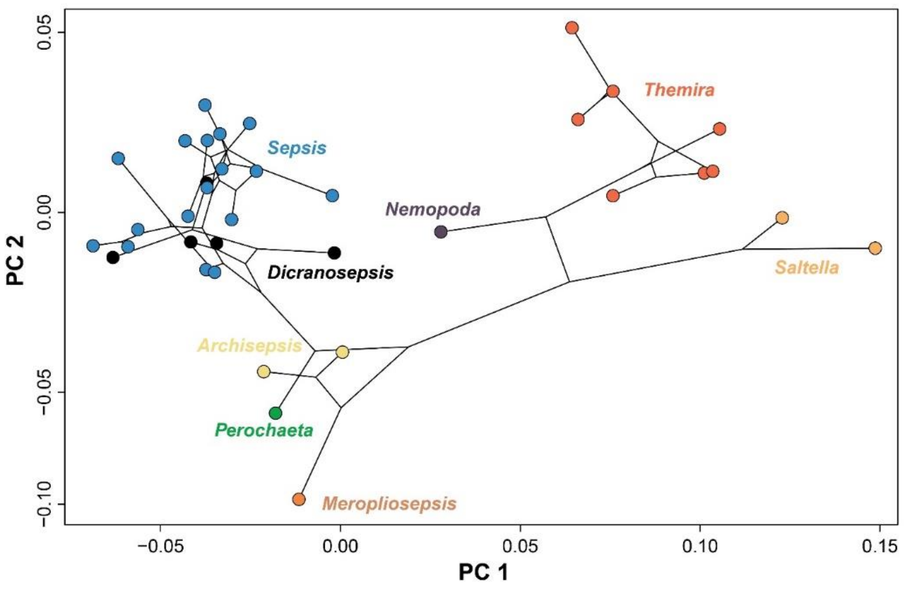

Fig. S3. Results of a phylogenetically aligned component analysis showing the phylogenetic relationship among sepsid flies.

Table S1. Procrustes ANOVA table for the analysis of fluctuating asymmetry in wing shape in *Sepsis punctum* and *Sepsis fulgens*. Allometric variation was statistically removed by taking the residuals of a multivariate regression of wing shape (i.e., Procrustes shape variables) on logarithmized centroid size prior to conducting the Procrustes ANOVA.

a) Sepsis punctum

	Df	SS	MS	F	Z	η^2	Pr(>F)
Individual	86	2.19E-01	2.55E-03	291.52	25.69	0.91	<.001
Side (left vs. right; directional asymmetry)	1	2.36E-04	2.36E-04	27.02	6.91	<.01	<.001
Individual×side (fluctuating asymmetry)	86	2.04E-02	2.38E-04	27.15	27.11	0.08	<.001
Residuals (individual×side×measurement)	174	1.52E-03	8.75E-06				

b) Sepsis fulgens

	Df	SS	MS	F	Z	η^2	Pr(>F)
Individual	95	1.83E-01	1.93E-03	221.77	29.51	0.90	<.001
Side (left vs. right; directional asymmetry)	1	1.27E-04	1.27E-04	14.62	6.96	<.01	<.001
Individual×side (fluctuating asymmetry)	95	1.86E-02	1.96E-04	22.52	20.88	0.09	<.001
Residuals (individual×side×measurement)	192	1.67E-03	8.70E-06				

Table S2. Slopes and correlations (including 95% REML-MVN confidence limits) for all common subspace comparisons using the eigenvectors of alternative matrices as basis for comparison. Because none of the matrices were full rank, we only used the minimum number of significant ranks in either of the three matrices used for the common subspace analysis (9 dimensions for comparisons between **D** and **G** matrices, 12 for all other comparisons).

a) eigenvectors used for comparison: phenotypic variation (**P**) in *S.fulgens*

*matrix compared to **D** in S. punctum*	*number of dimensions*	*slope (95% CI)*	*correlation (95% CI)*
**D** estimated in *S. fulgens*	9	0.65 (0.49,0.78)	0.95 (0.90,0.97)
**P** estimated in *S. punctum*	12	1.10 (0.98,1.21)	0.91 (0.86,0.94)
**P** estimated in *S. fulgens*	12	1.17 (1.03,1.28)	0.93 (0.88,0.95)
**G** estimated in *S. punctum*	9	1.27 (1.02,1.47)	0.91 (0.86,0.93)
**G** estimated in *S. fulgens*	9	1.03 (0.77,1.25)	0.90 (0.84,0.93)
**R** estimated in Sepsidae	12	1.27 (1.10,1.41)	0.88 (0.81,0.91)
**M** estimated in *D. melanogaster*†	12	0.78 (0.66,0.88)	0.87 (0.78,0.91)
**G** estimated in *D. melanogaster*†	12	0.79 (0.70,0.86)	0.89 (0.83,0.92)
**R** estimated in Drosophilidae†	12	0.60 (0.50,0.69)	0.62 (0.53,0.69)

b) eigenvectors used for comparison: developmental variation (**D**) in *S.fulgens*

*matrix compared to **D** in S. punctum*	*number of dimensions*	*slope (95% CI)*	*correlation (95% CI)*
**D** estimated in *S. fulgens*	9	0.85 (0.68,1.00)	0.98 (0.94,0.99)
**P** estimated in *S. punctum*	12	0.98 (0.88,1.06)	0.90 (0.86,0.93)
**P** estimated in *S. fulgens*	12	0.92 (0.83,1.00)	0.89 (0.85,0.92)
**G** estimated in *S. punctum*	9	1.25 (0.99,1.46)	0.90 (0.86,0.93)
**G** estimated in *S. fulgens*	9	0.66 (0.44,0.86)	0.88 (0.83,0.91)
**R** estimated in Sepsidae	12	1.01 (0.88,1.12)	0.81 (0.73,0.86)
**M** estimated in *D. melanogaster*†	12	0.68 (0.59,0.76)	0.90 (0.83,0.93)
**G** estimated in *D. melanogaster*†	12	0.77 (0.70,0.83)	0.93 (0.89,0.95)
**R** estimated in Drosophilidae†	12	0.68 (0.60,0.76)	0.79 (0.72,0.83)

c) eigenvectors used for comparison: phenotypic variation (**P**) in *S.punctum*

*matrix compared to **D** in S. punctum*	*number of dimensions*	*slope (95% CI)*	*correlation (95% CI)*
**D** estimated in *S. fulgens*	9	0.91 (0.71,1.07)	0.96 (0.90,0.98)
**P** estimated in *S. punctum*	12	1.48 (1.29,1.61)	0.96 (0.91,0.98)
**P** estimated in *S. fulgens*	12	1.18 (1.02,1.30)	0.94 (0.88,0.96)
**G** estimated in *S. punctum*	9	1.80 (1.50,2.04)	0.92 (0.86,0.95)
**G** estimated in *S. fulgens*	9	1.33 (1.05,1.55)	0.92 (0.84,0.95)
**R** estimated in Sepsidae	12	1.44 (1.22,1.60)	0.81 (0.73,0.87)
**M** estimated in *D. melanogaster*†	12	0.96 (0.81,1.09)	0.90 (0.82,0.94)
**G** estimated in *D. melanogaster*†	12	0.95 (0.83,1.04)	0.93 (0.87,0.96)
**R** estimated in Drosophilidae†	12	0.97 (0.81,1.09)	0.75 (0.67,0.81)

† Data from Houle et al. 2017 (1)

Table S3. AIC-based model comparison of reduced-rank factor analytic models with different numbers of dimensions for the respective matrix and species. The model selected for matrix comparisons is indicated with an asterisk (*).

	**D matrix** *S. punctum*		**D matrix** *S. fulgens*		**G matrix** *S. punctum*		**G matrix** *S. fulgens*		**P matrix** *S. punctum*		**P matrix** *S. fulgens*		**R matrix** Sepsidae	
**dimensions**	AIC	ΔAIC	AIC	ΔAIC	AIC	ΔAIC	AIC	ΔAIC	AIC	ΔAIC	AIC	ΔAIC	AIC	ΔAIC
1	39130.12	6156.7	42911.6	5539.4	248787.2	2987.6	110763.1	1245.4	64713.9	9920.1	69447.2	10133.7	133410.8	9284.8
2	37958.72	4985.3	41971.6	4599.4	247657.7	1858.1	110314.8	797.1	63112.4	8318.6	67135.9	7822.3	130261.6	6135.6
3	36729.25	3755.8	40793.9	3421.7	246803.6	1004	109997.5	479.8	60720.0	5926.2	65818.5	6504.9	128073.2	3947.2
4	36219.41	3246.0	40139.3	2767.1	246315.5	515.9	109802.5	284.8	59628.8	4835.0	64094.9	4828.3	126429.2	2303.2
5	35571.06	2597.6	39619.1	2246.8	246069.0	269.4	109674.7	157.0	58803.3	4009.4	63041.9	3775.3	125436.0	1310.0
6	34977.87	2004.5	38830.7	1458.5	245883.8	84.2	109587.5	69.8	57770.7	2976.8	62169.9	2903.3	124941.9	815.9
7	34392.05	1418.6	38316.7	944.5	245827.3	27.7	109549.8	32.1	56880.5	2086.6	61629.0	2362.4	124576.2	450.2
8	33937.94	964.5	37677.3	305.0	245808.5	8.9	109520.0	2.3	56288.8	1494.9	61200.4	1933.8	124378.2	252.2
9	33600.64	627.2	37372.2	0.0*	245799.6	0.0*	109517.7	0.0*	55754.4	960.5	60377.4	1110.8	124248.0	122.0
10	33300.69	327.3	no convergence		no convergence		109517.8	0.1	55460.1	666.3	60073.1	806.6	124077.7	39.3
11	33104.1	130.7					no convergence		55195.6	401.7	59814.2	547.7	124045.5	9.7
12	32973.41	0.0*							54968.6	174.7	59656.2	389.6	124126.0	0.0*
13	no convergence								54852.2	58.4	59445.0	178.4	124127.3	1.3
14									54793.9	0.0*	59313.6	47.0		
15									no convergence		59268.4	1.8*		
16											59266.6	0.0		

SI References

1. Houle D, Bolstad GH, van der Linde K, Hansen TF. Mutation predicts 40 million years of fly wing 363 evolution. Nature. 2017;548(7668):447-50.
